# Distribution characteristics and prognostic value of TIM-1 in patients with lung adenocarcinoma

**DOI:** 10.3389/fimmu.2025.1602868

**Published:** 2025-05-30

**Authors:** Junxu Wen, Wenhua Yun, Yu Chen, Xiaoyan Yin, Wenxing Cui, Minghao Yu, Xiangjiao Meng

**Affiliations:** Department of Radiation Oncology, Shandong Cancer Hospital Affiliated to Shandong First Medical University, Jinan, Shandong, China

**Keywords:** TIM-1, lung adenocarcinoma, prognosis, tumor microenvironment, tumor-draining lymph nodes, tertiary lymphoid structure

## Abstract

**Background:**

T-cell immunoglobulin and mucin domain-containing protein 1 (TIM-1) has been identified as a promoter of tumor cell viability, migration, and invasion. However, the precise role and distribution characteristics of TIM-1 within the tumor microenvironment (TME) remain critical areas of investigation.

**Methods:**

In this study, multiplex immunofluorescence (mIF) was performed on tissue slides from 126 patients with lung adenocarcinoma (LUAD) to investigate the distribution patterns of TIM-1 and the prognostic significance of three TIM-1 positive immune cell populations in both the primary tumor and tumor-draining lymph nodes (TDLN).

**Results:**

Compared to the primary tumor, TIM-1+CD8+T cells and TIM-1+B cells exhibited significantly greater density in the TDLN (p<0.0001, p<0.0001 respectively). In the primary tumor, lower TIM-1+B cell density was associated with longer overall survival (OS) (mOS, 84 vs. 54 months; p<0.0001, HR=2.574) and disease-free survival (DFS) (mDFS, 53.0 vs. 23.1 months; p=0.018, HR=1.721). In the TDLN, lower TIM-1+B cell density was also correlated with longer OS (mOS, not reached vs. 64.7 months; p=0.0019, HR=2.3502) and DFS (mDFS, 68.5 vs. 28.9 months; p=0.016, HR=1.707). Higher TIM-1+B cell density in the TDLN was associated with a lower proportion of mature tertiary lymphoid structures (TLS) (p=0.0009, r=-0.3990) and increased density of TIM-1+B cells in the tumor was linked to reduced CD8+ T cell density (p=0.016, r=-0.2788).

**Conclusions:**

Our findings confirm the immunosuppressive role of TIM-1+B cells in LUAD and suggest that TIM-1+B cells exert immune suppression by inhibiting TLS maturation and CD8+ T cell density. These findings highlight TIM-1+ B cells as a potential therapeutic target.

## Introduction

Non-small cell lung cancer (NSCLC) accounts for 85% of all lung cancer cases, which is the leading cause of cancer-related deaths worldwide. LUAD represents 50-60% of all NSCLC cases (1). Although advances in targeted therapy and immunotherapy have improved the treatment of NSCLC, the poor survival rates underscore the urgent need for further research and the development of more effective therapeutic regimens (2). Immune checkpoints, which negatively regulate immune responses, have become a focal point of cancer research, particularly immune checkpoint blockade (ICB). ICB targeting programmed cell death-1 (PD-1), its ligand (PD-L1), or cytotoxic T-lymphocyte-associated antigen 4 (CTLA-4) has shown durable anti-tumor effects in various cancers (3). Several ICBs have been approved as standard treatments for NSCLC patients, but unfortunately, only a limited number of patients respond to ICB therapy, and acquired resistance undermines the efficacy of ICB treatment (1).

A comprehensive analysis of the TME has become a major approach to identifying novel biomarkers and therapeutic targets for immunotherapy (4). Previous studies have primarily focused on immune regulatory molecules expressed on T cells. Surprisingly, a recent study established the pro-tumor effect of TIM-1 expressing on B cells in melanoma-bearing mouse model and the potential to limit tumor growth by blocking TIM-1 (5). TIM-1 is a type I cell surface glycoprotein encoded by HAVCR1 and is expressed by various immune cells, including T cells, B cells, and mast cells (6, 7). Except for immune cells, TIM-1 was also expressed by several kinds of tumor cells and the prognostic value of TIM-1 expression was demonstrated in other tumor types, such as liver carcinoma, pancreatic adenocarcinoma and renal cell carcinoma (8–11). In the context of lung cancer, TIM-1 expression levels have been demonstrated to correlate with patient prognosis and modulate tumor cell activity (12). However, whether TIM-1 is expressed on immune cells and the functional roles of TIM-1+ immune cells remain to be further elucidated. In addition, the distribution and prognostic significance of TIM-1-positive immune cells in the TME remain areas requiring further investigation. With the further studies of tumor immunity, it is unquestionable that TDLN also play a indispensable role in anti-tumor immune response (13). The role of TIM-1-positive immune cells in the TDLN of NSCLC patients remains poorly understood.

CD8+ T cells are crucial players in the anti-tumor immune response and are associated with better prognosis in LUAD patients (14). The density of CD8+T cell is one of the main determinants of the response to anti-tumor immunotherapies (15, 16). Enhancing CD8+ T cell density remains a major challenge in tumor immunology research. Additionally, TLS, especially mature TLS, are important participants in the anti-tumor immune response and are linked to better responses to immunotherapy (17, 18). The factors that regulate TLS maturation are still not fully understood.

In this study, we employed mIF to examine the distribution patterns of TIM-1 and assess the prognostic value of three TIM-1-positive immune cell populations in both the primary tumor and TDLN. We observed that TIM-1+B cells were associated with poor prognosis in LUAD patients, both in the primary tumor and the TDLN. TIM-1+B cells in the TDLN may impair TLS maturation, while TIM-1+B cells in the primary tumor may reduce CD8+ T cell density. These findings suggest that TIM-1+B cells could represent a potential target for immunotherapy in LUAD patients.

## Methods

### Patient cohort

**Figure 1:**
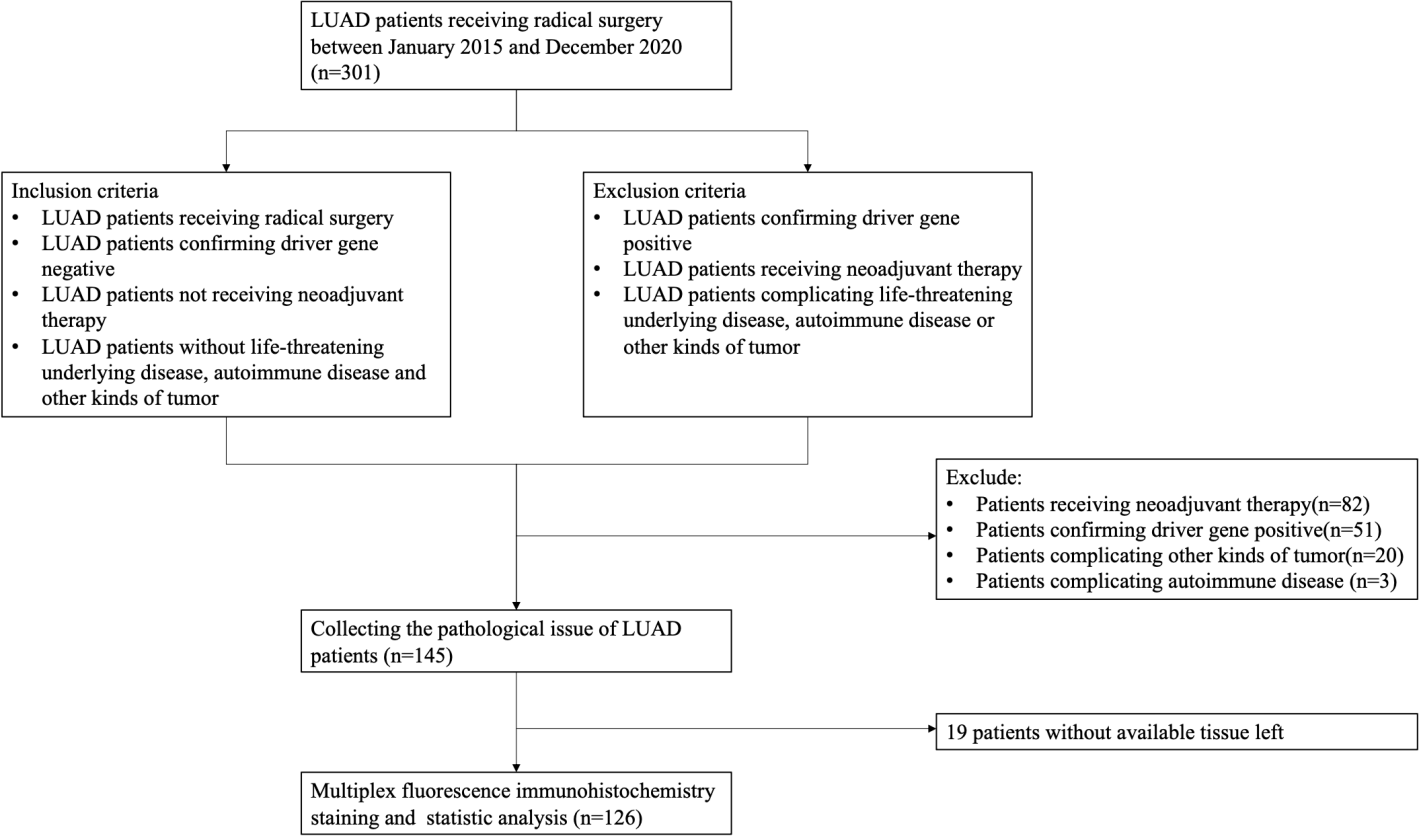
Enrollment flow diagram. A total of 301 patients with lung adenocarcinoma (LUAD) were screened between January 2016 and December 2020. Finally, 126 patients were selected for the further staining and analysis.

This study retrospectively enrolled LUAD patients who received radical surgery between January 2016 and December 2020. Based on the molecular profiling results, we exluded patients with confirming drive gene positive (EGFR, ALK, KRAS, ROS1, MET). In addition, patients receiving any neoadjuvant therapies (chemotherapy, radiotherapy, immunotherapy or targeted therapy before surgery) were also excluded. The details of inclusion criteria and exclusion criteria and enrollment flow diagram were displayed at **Figure 1**. Clinical information was collected from records in the electric medical system. The pathological staging of patients was re-evaluated in accordance with the 9th edition of the American Joint Committee on Cancer (AJCC) Staging System. OS was calculated from the surgical date until the last documented follow-up or all-cause mortality. DFS referred to the duration between surgery and the earliest occurrence of tumor recurrence, disease progression, or death from any cause. This study was approved by the Ethics Review Board of Shandong Cancer Hospital Affiliated to Shandong First Medical University.

### Multiplex immunofluorescence staining

**Figure 2:**
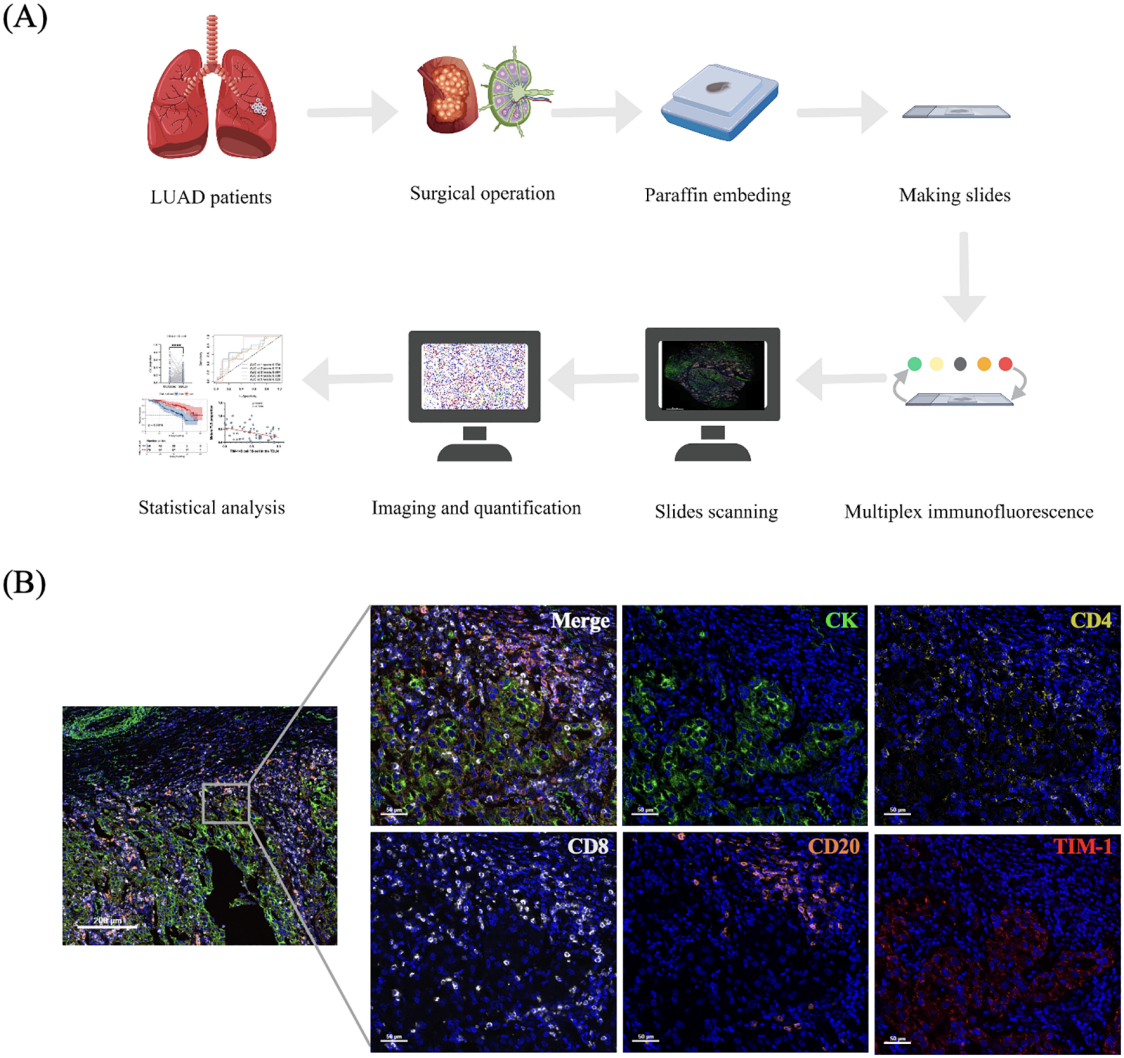
Multiplex immunofluorescence staining on LUAD slides. **(A)** Schematic illustration of the experimental design and analytical approaches employed in this research. **(B)** The staining results of panel 1 (DAPI, CK, CD4, CD8, CD20, TIM-1).

Formalin-fixed paraffin-embedded (FFPE) tumor tissues and TDLN tissues from LUAD patients were collected to generate 4μm slides (**Figure 2A**). All paraffin blocks used in this study were derived from postoperative pathological tissues of patients. Multiplex immunofluorescence staining was performed using the Akoya Opal Kit (Akoya Biosciences, USA; NEL871001KT) in the complete histological sections. Each slide was incubated overnight at 55°C, followed by dewaxing with xylene and rehydration through a graded series of ethanol solutions. Antigen retrieval was performed using ethylenediaminetetraacetic acid (EDTA, pH 8.0) and sodium citrate buffer (pH 6.0) at 95°C for 20 minutes. After cooling to room temperature, antigen blocking was carried out with 40% goat serum. Subsequently, each slide was incubated with specific primary antibodies, Polymer HRP, and Opal fluorophore solution sequentially. Between each incubation step, the slides were washed with TBST (5 minutes × 3). A total of five rounds of staining were performed. The primary antibodies used in panel 1 were CK (ZSGB-BIO, ZM-0069, 1:200), CD4 (ZSGB-BIO, ZM-0418, 1:500), CD20 (Proteintech, 60271-1Ig, 1:1000), TIM-1 (ThermoFisher, PA5-79345,1:500), and CD8 (Abcam, ab199016, 1:400) in sequence (**Figure 2B**). The fluorescent dyes corresponding to the primary antibodies are 480, 570, 690, 620, and 780 respectively. All of them are used at a dilution ratio of 1:150. The primary antibodies used in panel 2 were CD20 (Proteintech, 60271-1Ig, 1:1000), CD21 (ABclonal, A8407, 1:100), and CD23 (Abcam, ab92495, 1:800). The fluorescent dyes corresponding to the primary antibodies are 480, 690, and 520 respectively. All of them are used at a dilution ratio of 1:150. Finally, DAPI solution was applied to label the cell nuclei. Based on previous studies (19) and the results from panel 2, TLS were classified into three distinct maturation stages: Early TLS (E-TLS, CD20+CD21−CD23−), Primary Follicle-like TLS (PFL-TLS, CD20+CD21+CD23−), and Secondary Follicle-like TLS (SFL-TLS, CD20+CD21+CD23+). Only CD23-positive TLS (SFL-TLS) were considered mature TLS.

### Imaging and quantification

The stained slides were scanned using the Vectra/Polaris Scanner System (3.0.3, Akoya Biosciences) to obtain images for subsequent analysis. Due to technical limitations, panoramic analysis of whole-slide stained images was not feasible. Therefore, in previous studies (20–23), a defined number of regions of interest (ROIs) were selected for subsequent analysis. We randomly selected five 1 × 1 fields (931 μm × 698 μm) from each slide, which were then imported into the inForm 2.4.8 system (PerkinElmer, USA) for further processing and analysis. First, the tumor and stromal compartments were visually identified using epithelial cell marker (CK) signals across at least 10 images to train a tissue classifier. Subsequently, all images underwent segmentation using this classifier, achieving an accuracy exceeding 95% (>98% in our study). Third, since the images contained heterogeneous cell populations with distinct nuclear and cellular morphologies, we performed adaptive cell segmentation by integrating nuclear (DAPI), membrane (CD4, CD8, CD20, TIM-1), and cytoplasmic (CK) signals. Finally, similar to tissue segmentation, we trained a single-marker phenotype classifier by manually annotating ≥20 positive and ≥20 negative cells per marker. All images were then processed using this classifier, which achieved >95% accuracy (exceeding 99% for each cell type in our study). The classification results of all tissue and cell classifiers were visually validated by at least two independent researchers to further ensure accuracy. Upon completion of these three steps, the inForm software generated cell phenotype data including the cell counts of different cell types (tumor cell, CD4+T cell, CD8+T cell, B cell, TIM-1+CD4+T cell, TIM-1+CD8+T cell and TIM-1+B cell) within the different regions. Based on the previous studies (18, 24), the abundance of TLSs was scored as 0, 1, 2 according to the number of TLSs. A score of 0 indicated the absence of TLS. A score of 1 indicated less than three TLS. A score of 3 indicated three or more TLS. Only patients with score 2 and mature TLS were identified as TLS (+). Patients with score 0 or 1 and patients without mature TLS were TLS (–). The evaluation of TLS, including quantitative analysis and qualitative characterization, was conducted using complete whole-slide digital images. TLSs were independently assessed by two pathologists who were blinded to the patients’ clinical information.

### Statistical analysis

All the statistical analyses were performed by SPSS 27.0 (IBM, USA), GraphPad Prism 10.3.1 (464) and R software. The cellular density was calculated by normalizing the cell counts against the total number of cells in each sample. Density of A = n (A)/n (total cells). According to the results of the Kolmogorov-Smirnov test, all cell phenotype parameters did not follow a normal distribution. Therefore, the differences between two groups were analyzed using the Mann-Whitney U test, while the differences between paired groups were assessed using the Wilcoxon signed-rank test. ANOVA was employed to compare multiple groups. Time-dependent receiver-operating characteristic (ROC) curves were used to determine the optimal cutoff values (25), which were then used to categorize patients into high-density and low-density groups. Survival analysis was performed using the Kaplan-Meier method, and the log-rank test was employed to assess disparities among groups. Cox proportional hazards regression models were used to evaluate the independent prognostic value of each parameter. The correlation between different cell types was assessed using the Spearman rank correlation coefficient. A p-value of less than 0.05 was considered statistically significant.

## Results

### Patient clinicopathological characteristics

A total of 126 patients with LUAD who underwent radical surgery between January 2016 and December 2020 were enrolled. Of these, 55.6% were diagnosed with lymph node metastasis (LNM) based on postoperative pathology. Among patients with LNM, 34.3% had N1 stage and 65.7% had N2 stage (**Table 1**). Of the cohort, 63 patients (50.0%) were male and 63 patients (50.0%) were female. The median age of the patients was 58.14 years, with a range from 37 to 76 years, and 67 patients (53.2%) were aged ≥60 years. The majority of patients had no smoking history (65.9%), were staged as T2 (50.8%), were classified as stage I (41.3%), and had moderate differentiation (71.4%). The median follow-up time was 63.9 months (95% CI: 61.05–66.81). The mOS was 79.0 months (95% CI: 64.47–93.53), and the mDFS was 53.0 months (95% CI: 35.02–71.05).

**Table 1:** The clinicopathological characteristics of enrolled lung adenocarcinoma (LUAD) patients.

Variable	N=126	Percentage
Age		
<60	59	46.8%
≥60	67	53.2%
Gender		
Male	63	50.0%
Female	63	50.0%
Smoking history		
No	83	65.9%
Yes	43	34.1%
T stage		
T1	52	41.3%
T2	64	50.8%
T3	10	7.9%
Lymphatic metastasis		
N0	56	44.4%
N1	24	19.1%
N2	46	36.5%
Stage		
I	52	41.3%
II	30	23.8%
III	44	34.9%
Differentiation		
Poor	28	22.2%
Moderate	90	71.4%
Well	8	6.4%

### TIM-1+cells mainly distributed in the tumor draining lymph nodes

**Figure 3:**
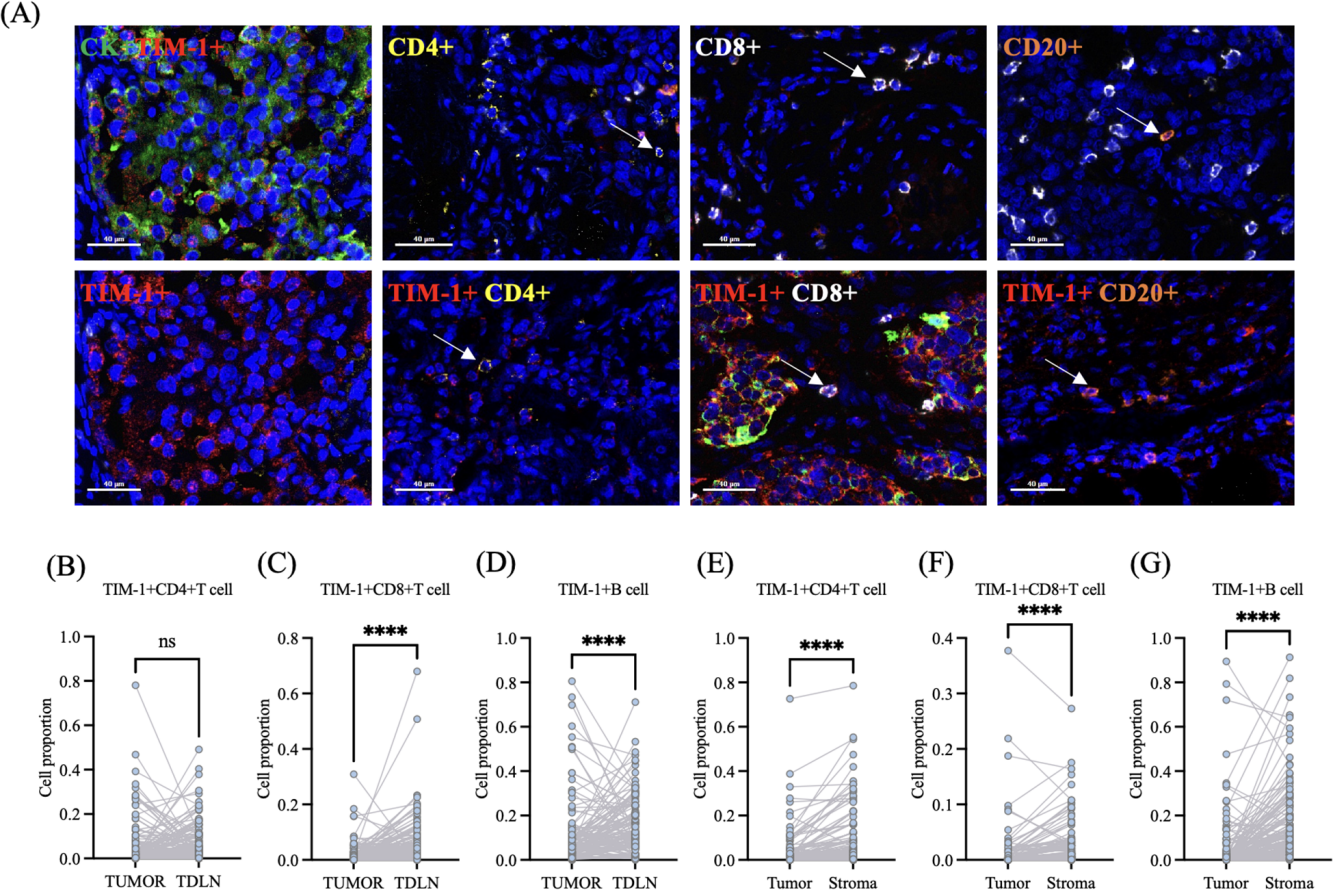
The distribution characteristic of TIM-1 in the tumor microenvironment. **(A)** TIM-1 was distributed in tumor cells, CD4+T cells, CD8+T cells and B cells. **(B)** There was no difference of the proportion of TIM-1+CD4+T cell among all cell counts in the tumor primary lesion and TDLN. **(C)** TIM-1+CD8+T cell mainly distribute in the TDLN. **(D)** TIM-1+B cell mainly distribute in the TDLN. **(E)** In the tumor primary lesion, TIM-1+CD4+T cell mainly distribute in the stroma. **(F)** In the tumor primary lesion, TIM-1+CD8+T cell mainly distribute in the stroma. **(G)** In the tumor primary lesion, TIM-1+B cell mainly distribute in the stroma. ns p > 0.05; ****P ≤ 0.0001. ns: not significant.

A previous study demonstrated that TIM-1 expression was associated with poor prognosis in NSCLC patients (12). To further explore the distribution characteristics of TIM-1 in the TME, mIF was performed on human LUAD primary lesions and paired TDLN. Consistent with previous findings (12), TIM-1 was extensively expressed in lung adenocarcinoma cells (**Figure 3A**). Additionally, the density of TIM-1+CD4+ T cells, TIM-1+CD8+ T cells, and TIM-1+B cells in both the tumor primary lesion and TDLNs was observed (**Figure 3A**). The densities of these TIM-1-positive cell types were calculated for further analysis. Paired Wilcoxon signed-rank test was used to explore the difference of densities of these TIM-1-positive cell types between tumor and TDLN or between tumor and stroma. There was no significant difference in the density of TIM-1+CD4+ T cells between the tumor and TDLN (p = 0.1484) (**Figure 3B**). However, compared to the tumor primary lesion, TIM-1+CD8+ T cells and TIM-1+B cells were more infiltrated in the TDLNs (p < 0.0001, p < 0.0001, respectively) (**Figures 3C, D**). Based on CK expression, the tumor primary lesions were classified into tumor and stroma regions using the Vectra Polaris imaging system. Compared to the tumor, TIM-1+CD4+ T cells, TIM-1+CD8+ T cells, and TIM-1+B cells exhibited higher density in the stroma (p < 0.0001, p < 0.0001, p < 0.0001, respectively) (**Figures 3E–G**).

### Density of TIM-1+B cells determines poor prognosis in patients with LUAD

**Figure 4:**
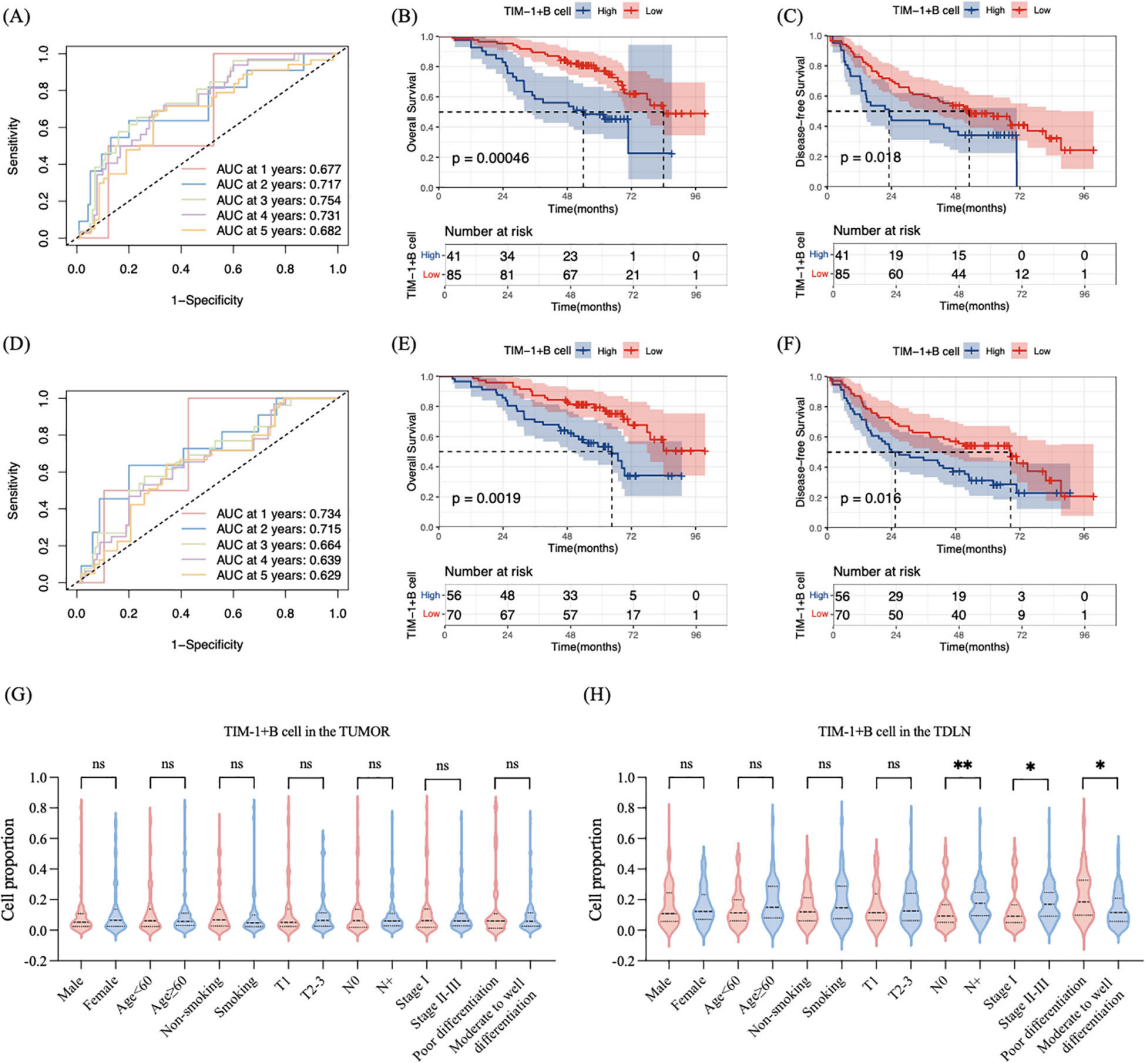
TIM-1+B cell was associated with poor prognosis. **(A)** Time-dependent ROC curve for TIM-1+B cell in the tumor primary lesion. **(B)** High density of TIM-1+B cell in the tumor primary lesion was associated with shorter OS. **(C)** High density of TIM-1+B cell in the tumor primary lesion was associated with shorter DFS. **(D)** Time-dependent ROC curve for TIM-1+B cell in the TDLN. **(E)** High density of TIM-1+B cell in the TDLN was associated with shorter OS. **(F)** High density of TIM-1+B cell in the TDLN was associated with shorter DFS. **(G)** The density of TIM-1+B cell in the tumor primary lesion was not related to the age, sex, smoking history, T stage, N stage, tumor stage and tumor differentiation. **(H)** The high density of TIM-1+B cell in the TDLN was related to the lymph node metastasis, later tumor stage and poor differentiation. *P ≤ 0.05 **P ≤ 0.01; ns, not significant.

Previous studies have shown that TIM-1 plays a pro-tumor and immunosuppressive role. To further investigate the prognostic value of three types of TIM-1-positive immune cells (TIM-1+CD4+ T cells, TIM-1+CD8+ T cells, and TIM-1+B cells) in patients with LUAD, we conducted a comprehensive analysis. Initially, we determined the optimal cutoff values for the density of each cell type using time-dependent ROC curves (**Figures 4A, D**, **Supplementary Figure S3**). Higher area under the ROC curve (AUC) values indicates better marker performance. The cutoff values for the density of each cell type are listed in **Supplementary Table S1**. In both the tumor primary lesion and TDLNs, TIM-1+CD4+ T cells and TIM-1+CD8+ T cells did not show prognostic value for predicting OS or DFS in LUAD patients (**Supplementary Figure S1**). However, higher density of TIM-1+B cells in both the tumor primary lesion and TDLN was associated with worse prognosis. In the tumor primary lesion, patients with lower density of TIM-1+B cells had longer OS (mOS, 84 vs. 54 months; p < 0.0001, HR = 2.574, 95% CI 1.356 to 4.889) (**Figure 4B**) and DFS (mDFS, 53.0 vs. 23.1 months; p = 0.018, HR = 1.721, 95% CI 1.035 to 2.862) (**Figure 4C**). In the TDLN, patients with lower density of TIM-1+B cells also had longer OS (mOS, not reached vs. 64.7 months; p = 0.0019, HR = 2.350, 95% CI 1.323 to 4.172) (**Figure 4E**) and DFS (mDFS, 68.5 vs. 28.9 months; p = 0.016, HR = 1.707, 95% CI 1.081 to 2.696) (**Figure 4F**). These findings suggest that TIM-1+B cells may exert a pro-tumor effect and are associated with poor prognosis, both in the tumor primary lesion and the TDLN. To validate our findings, we enrolled 35 patients as a validation cohort. In the validation cohort, high density of TIM-1+B cell in the tumor and TDLN was correlated with shorter OS (p=0.029, p=0.01 respectively) (**Supplementary Figures S2B, D**). Patients with high density of TIM-1+B cell in the TDLN showed shorter DFS (p=0.0079) (**Supplementary Figure S2C**). The correlation between TIM-1+ B cell density and DFS was not statistically significant (p=0.059), potentially owing to the small cohort size (n=35) (**Supplementary Figure S2A**). Subsequently, we examined the relationship between TIM-1+B cell density and clinicopathological characteristics in LUAD patients by the Mann-Whitney U test. In the tumor primary lesion, TIM-1+B cell density was not associated with age (p = 0.9137), sex (p = 0.4591), smoking history (p = 0.2852), T stage (p = 0.9537), N stage (p = 0.9075), tumor stage (p = 0.6400), or tumor differentiation (p = 0.8040) (**Figure 4G**). In the TDLN, patients with lymph node metastasis had higher density of TIM-1+B cells than those without lymph node metastasis (p = 0.0489). Additionally, patients in stages II-III had higher density of TIM-1+B cells compared to patients in stage I (p = 0.0052). Patients with poorly differentiated tumors had higher TIM-1+B cell density than those with moderate to well-differentiated tumors (p = 0.0073). However, TIM-1+B cell density in the TDLN was not associated with age (p = 0.0629), sex (p = 0.7753), smoking history (p = 0.3578), or T stage (p = 0.5378) (**Figure 4H**). These findings suggest that the density level of TIM-1+B cells in the TDLN may increase with tumor progression. Patients with high-malignancy tumors may exhibit higher density of TIM-1+B cells in the TDLN. We further validated the prognostic value of TIM-1+B cells using the Cox regression model (**Table 2**). Univariate analysis revealed that T stage (p = 0.002), lymphatic metastasis (p = 0.007), tumor stage (p = 0.005), CD8+ T cell density in the tumor (p = 0.001), B cell density in the tumor (p = 0.001), TIM-1+B cell density in the tumor (p = 0.001), and TIM-1+B cell density in the TDLN (p = 0.003) were significantly associated with OS in LUAD patients. Multivariate analysis indicated that T stage (p = 0.013), tumor stage (p = 0.033), and TIM-1+B cell density in the tumor (p < 0.001) were independently associated with OS. These results suggest that TIM-1+B cell density in the tumor can serve as an independent prognostic predictor for LUAD patients.

**Table 2:** Univariate analyses and multivariate analyses of prognostic markers for overall survival (OS) in lung adenocarcinoma (LUAD).

Variable	Univariate analysis		Multivariate analysis	
	HR (95% CI)	P	HR (95% CI)	P
Age				
<60 vs ≥60	1.570 (0.886-2.782)	0.123		
Gender				
Male vs Female	1.376 (0.746-2.538)	0.306		
Smoking history				
No vs Yes	0.894 (0.498-1.605)	0.707		
T stage				
T1 vs T2-3	3.441 (1.589-7.449)	0.002	2.941 (1.265-6.889)	0.013
Lymphatic metastasis				
No vs Yes	2.460 (1.277-4.738)	0.007	0.673 (0.173-2.623)	0.568
Stage				
I vs II-III	2.461 (1.318-4.595)	0.005	4.912 (1.133-21.307)	0.033
Differentiation				
Poor vs Moderate to well	0.586 (0.319-1.077)	0.085		
Tumor CD4+T cell				
Low vs High	1.830 (0.985-3.399)	0.056		
Tumor CD8+T cell				
Low vs High	0.358 (0.193-0.663)	0.001	0.499 (0.240-1.037)	0.063
Tumor B cell				
Low vs High	3.449 (1.625-7.332)	0.001	0.876 (0.446-1.719)	0.700
Tumor TIM-1+B cell				
Low vs High	2.656 (1.505-4.688)	0.001	3.970 (1.990-7.920)	<0.001
TDLN CD8+T cell				
Low vs High	0.608 (0.338-1.092)	0.096		
TDLN TIM-1+B cell				
Low vs High	2.391 (1.355-4.217)	0.003	1.474 (0.754-2.883)	0.256

### High TIM-1+B cell/B cell in TDLN hinder the mature of TLS

**Figure 5:**
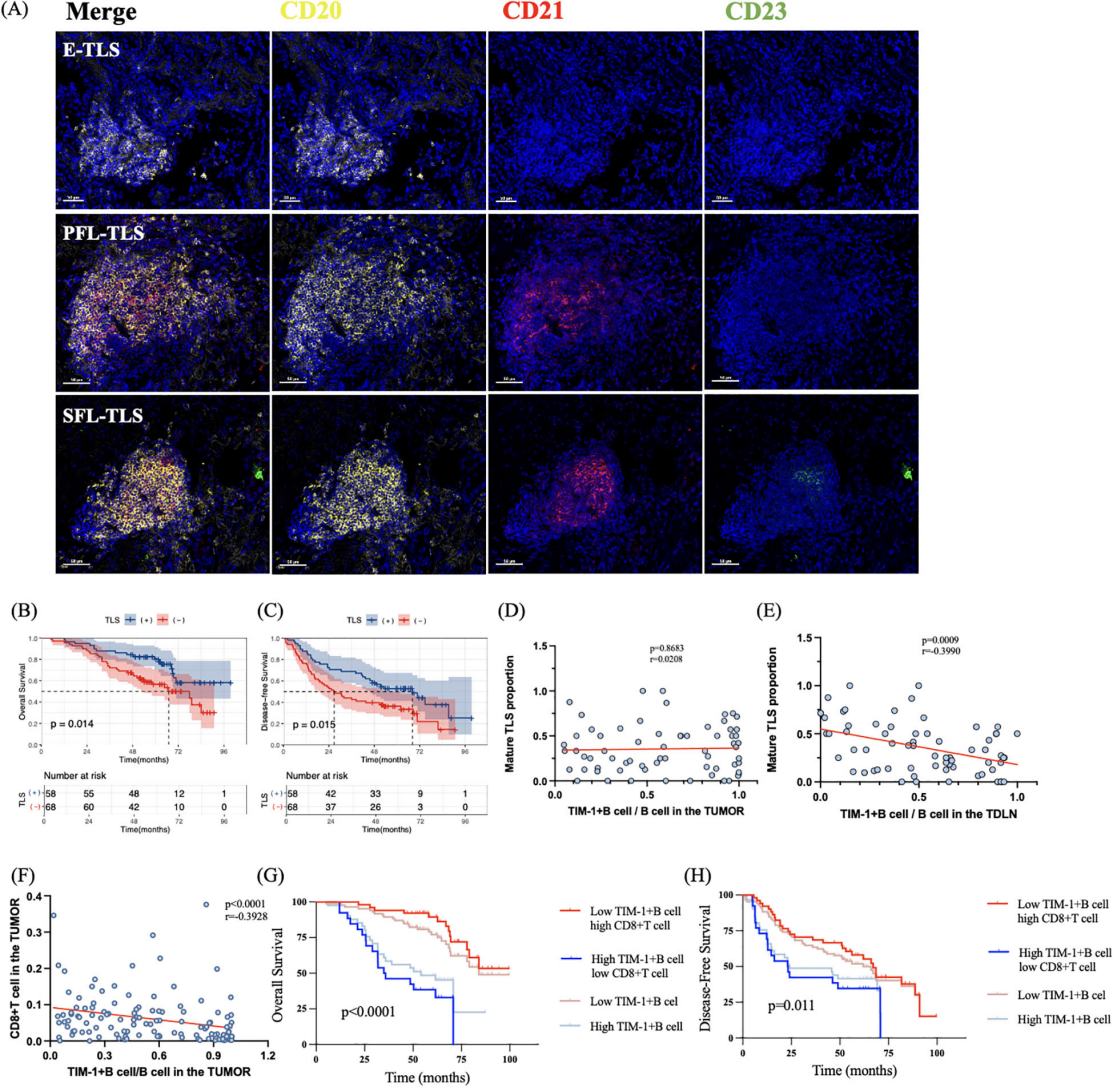
The mechanisms by which TIM-1+B cell affect the prognosis. **(A)** The staining images of TLSs in three different maturation states. **(B)** TLS (+) patients showed better OS than TLS (–) patients. **(C)** TLS (+) patients showed better DFS than TLS (–) patients. **(D)** There was no correlation between TIM-1+B cell/B cell in the tumor and mature TLS proportion. **(E)** High TIM-1+B cell/B cell in the TDLN was associated with low mature TLS proportion. **(F)** High proportion of TIM-1+B cell/B cell in the tumor was associated with low density of CD8+T cell. **(G, H)** Combination of TIM-1+B cell and CD8+T cell can better predict the OS and DFS of LUAD patients.

TLS, organized aggregates of immune cells within non-lymphoid tissues, play a critical role in tumor immune response and prognosis, with B cells being one of the main components of TLS (26–28). We investigated the correlation between TIM-1+B cells and TLS to explore the underlying mechanisms through which TIM-1+B cells influence prognosis. Panel 2 (DAPI, CD20, CD21, CD23) was used to quantify and classify TLS. TLS were categorized as early TLS (E-TLS, CD20+CD21–CD23–), primary follicle-like TLS (PFL-TLS, CD20+CD21+CD23–), and secondary follicle-like TLS (SFL-TLS, CD20+CD21+CD23+) (**Figure 5A**). Only CD23-positive TLS (SFL-TLS) were considered mature TLS. Since mature TLS are the primary functional TLS (29), focusing solely on the quantity of TLS does not reflect both their number and maturity. Therefore, we categorized patients into TLS (+) and TLS (−) groups by considering both the quantity and maturity of TLS (as described in the Methods section) (24). Patients were classified into TLS (+) and TLS (−) groups based on the criteria outlined in the “Imaging and Quantification” section. TLS (+) patients showed longer OS (mOS, not reached vs. 66.97 months; p = 0.014, HR = 0.4889, 95% CI 0.2808 to 0.8513) (**Figure 5B**) and DFS (mDFS, 68.2 vs. 26.8 months; p = 0.015, HR = 0.5767, 95% CI 0.3696 to 0.8999) compared to TLS (−) patients (**Figure 5C**). However, no significant correlation was found between the density of TIM-1+B cells within B cells and the number of TLS (**Supplementary Figure S3**). We then analyzed the correlation between the proportion of TIM-1+B cells within B cells and the proportion of mature TLS in patients with three or more TLSs. The correlation was assessed using the Spearman rank correlation coefficient. No correlation was observed between TIM-1+B cell/B cell density in the tumor primary lesion and TLS maturity (p = 0.8683, r = 0.0208) (**Figure 5D**). However, a higher TIM-1+B cell/B cell ratio in the TDLN was associated with a lower proportion of mature TLS (p = 0.0009, r = −0.3990, 95% CI −0.5895 to −0.1666) (**Figure 5E**). These results suggest that a higher proportion of TIM-1+B cells within B cells in the TDLN may impede TLS maturation, thereby contributing to a poorer prognosis.

### TIM-1+B cell decrease the density of CD8+T cell to affect the prognosis of LUAD patients

We further explored whether TIM-1+B cells could affect the density of T cells. We found that higher density of TIM-1+B cells in the tumor was associated with a reduced density of CD8+ T cells in the tumor (p = 0.016, r = −0.2788, 95% CI −0.3598 to −0.0233)(**Supplementary Figure S3C**). In addition, higher proportion of TIM-1+B cell/B cell in the tumor was associated with a reduced density of CD8+ T cells in the tumor (p <0.0001, r = −0.3928, 95% CI −0.5349 to −0.2290) (**Figure 5F**). However, TIM-1+B cells in the tumor were not related to the density of CD4+ T cells in the tumor (p = 0.0924, r = 0.1506, 95% CI −0.0302 to 0.3218) (**Supplementary Figure S3D**). Additionally, there was no significant relationship between TIM-1+B cells in the TDLN and the density of CD4+ T cells (p = 0.4258, r = 0.0712, 95% CI −0.1102 to 0.2479) or CD8+ T cells (p = 0.1094, r = −0.1433, 95% CI −0.3152 to 0.0376) in the tumor (**Supplementary Figures S3E, F**). These findings suggest that TIM-1+B cells in the tumor may contribute to poor prognosis in LUAD patients by reducing the density of CD8+ T cells in the tumor. In this study, higher density of CD8+ T cells correlated with better OS (mOS, not reached vs. 59.5 months; p < 0.0001, HR = 0.356, 95% CI 0.1892 to 0.5952) and DFS (mDFS, 67.1 vs. 35.2 months; p = 0.041, HR = 0.632, 95% CI 0.3977 to 1.004). Therefore, we further investigated the prognostic value of combining TIM-1+B cells in the tumor with CD8+ T cell density. The results indicated that this combination provided a better prediction of OS (p = 0.0022) and DFS (p = 0.011) in LUAD patients compared to TIM-1+B cells alone. Patients with low TIM-1+B cell and high CD8+ T cell density showed better OS and DFS than those with low TIM-1+B cell density. Conversely, patients with high TIM-1+B cell and low CD8+ T cell density exhibited poorer OS and DFS than those with high TIM-1+B cell density (**Figures 5G, H**).

## Discussion

Previous studies have primarily focused on TIM-1 expression in tumor cells, showing that TIM-1 acts as a pro-tumor factor and is associated with poor prognosis. In this study, we employed mIF to explore the distribution characteristics of TIM-1+cells in the TME and assess the prognostic value of different TIM-1-positive immune cells. The results revealed that, in addition to tumor cells, TIM-1 is also expressed on CD4+ T cells, CD8+ T cells, and B cells within both the tumor primary lesion and TDLN. Notably, TIM-1+CD8+ T cells and TIM-1+B cells were primarily located in the TDLN rather than in the tumor primary lesion. Within the tumor primary lesion, TIM-1+CD4+ T cells, TIM-1+CD8+ T cells, and TIM-1+B cells were predominantly found in the stromal area. These findings indicate that TIM-1+ immune cells are predominantly localized within TDLNs, establishing a foundation for investigating their cellular origins. High density of TIM-1+B cells was associated with poor OS and DFS, both in the tumor primary lesion and TDLN. Additionally, patients with lymph node metastasis, advanced tumor stage and poorly differentiated tumors exhibited higher TIM-1+B cell density in the TDLN. These results demonstrate that TIM-1+ B cells are significantly associated with adverse clinicopathological characteristics. Multivariate analyses identified TIM-1+B cell density in the tumor primary lesion as an independent prognostic predictor for LUAD patients.

In prior studies, TIM-1 has been linked to poor prognosis in various cancers (8–12, 30, 31). In the tumor cells, TIM-1 could improve the cell viability and the abilities of migration and invasion to cause a poor prognosis (12). Although some studies have demonstrated the expression of TIM-1 on T cell and B cell (6, 7, 32), previous research primarily focused on total TIM-1 expression, and the functional role of TIM-1 on immune cells remains inadequately explored. In this study, we investigated the distribution of TIM-1 on CD4+ T cells, CD8+ T cells, and B cells in the tumor primary lesion and TDLN for the first time. Our findings show that TIM-1+CD8+ T cells and TIM-1+B cells are predominantly found in the TDLN. Moreover, this is the first study to examine the prognostic value of TIM-1+CD4+ T cells, TIM-1+CD8+ T cells, and TIM-1+B cells in LUAD patients. We demonstrated that neither TIM-1+CD4+ T cells nor TIM-1+CD8+ T cells were correlated with the prognosis of LUAD. However, we confirmed that TIM-1+B cells were associated with poor prognosis, both in the tumor primary lesion and in the TDLN. This finding aligns with previous research on mouse models of B16F10 melanoma, where TIM-1+B cells promoted tumor growth and were linked to poor prognosis (5). Interestingly, this study also identified TIM-1 as a B-cell-specific checkpoint molecule, suggesting that TIM-1 expression on B cells may serve as a potential therapeutic target for tumor immunotherapy (33). Immune checkpoints in the B cells also play critical roles in the TME and deserve more attention in the future studies (34).

Our study bridges a significant gap in LUAD research by highlighting the prognostic significance of TIM-1+B cells. We also found that TIM-1+B cells in the TDLN correlated with lymph node metastasis, advanced tumor stage, and poorer tumor differentiation, thereby confirming their adverse prognostic effect. After validating the prognostic value of TIM-1+B cells, we explored potential mechanisms through which TIM-1+B cells influence LUAD prognosis. TLS, organized aggregates of immune cells in non-lymphoid tissues, play a crucial role in the tumor immune response (35). Since B cells are a key component of TLS (26), we first assessed the correlation between TIM-1+B cells and TLS. We found that TLS+ LUAD patients had better DFS and OS, consistent with prior studies (36). Notably, the density of TIM-1+B cells in the TDLN correlated with a reduced proportion of mature TLS. We hypothesize that TIM-1+B cells in the TDLN may negatively affect prognosis by inhibiting the maturation of TLS. In addition, CD8+ T cells are vital for anti-tumor immunity and correlate with better prognosis in NSCLC (37, 38). We then investigated whether TIM-1+B cells influence the density of CD8+ T cells in the tumor. The results indicated that high density of TIM-1+B cells in the tumor primary lesion was associated with reduced density of CD8+ T cells. Based on this, we hypothesize that TIM-1+B cells in the tumor primary lesion contribute to poor prognosis by hindering the density of CD8+ T cells. Interestingly, when considering the combination of TIM-1+B cells and CD8+ T cells, this combination provided superior prognostic value compared to TIM-1+B cells alone. Our findings suggest that both TIM-1+B cells and the combination of TIM-1+B cells with CD8+ T cells could serve as valuable prognostic markers for LUAD patients. TIM-1+B cells seem to limit CD8+ T cell density and hinder the maturation of TLS, indicating that TIM-1+B cells could be a promising target for immunotherapy in LUAD. Reducing TIM-1+B cell density may help enhance CD8+ T cell density and improve TLS maturation, which could potentially boost the efficacy of immunotherapy. A recent study showed that TIM-1 was higher expressed on LUAD patients who did not sensitive to ICBs (39). We propose that it is necessary to explore the relationship between TIM-1+B cell and the response to ICBs in the future studies. Integrating findings from our study with previous research (31), TIM-1 has been demonstrated to promote tumorigenesis in both tumor cells and B cells, and is associated with poor prognosis. Therefore, TIM-1 inhibitors represent promising anti-tumor agents. Furthermore, bispecific antibodies (bsAbs), a novel anticancer therapy, can simultaneously target two antigens. Specifically, a BsAb targeting both TIM-1 and CD20 could achieve selective depletion of TIM-1+ B cells, potentially improving clinical outcomes in LUAD patients.

However, there are certain limitations to this study that should be acknowledged. First, the relatively small sample size limits the generalizability of our findings. Therefore, future studies should involve larger, more heterogeneous cohorts to confirm our results. Furthermore, in future studies, enrolling larger multi-center cohorts and developing integrated prognostic prediction models will significantly advance precision oncology by enabling tailored therapeutic strategies based on individual immune and molecular profiles. Second, this research does not delve into the underlying mechanisms or specific signaling pathways that mediate the effects of TIM-1+B cells in LUAD. Future investigations should focus on exploring these mechanisms in depth. Third, due to the inherent limitations of mIF staining in the number of primary antibodies that can be simultaneously applied, our study included a relatively narrow spectrum of immune cell types. TIM-1 is expressed on both effector and regulatory immune cells, and its underlying mechanisms require further investigation. In future studies, flow cytometry or single-cell RNA sequencing (scRNA-seq) will be necessary to investigate whether TIM-1 is expressed in other immune cell subsets (e.g., macrophages, neutrophils) and to explore how TIM-1^+^ B cells may influence the infiltration or functional states of other immune populations within the TME. Lastly, due to the limited number of patients who underwent immunotherapy in this study, we were unable to assess the prognostic value of TIM-1+B cells in predicting response to immunotherapy. Future research will include more patients receiving immunotherapy to examine this aspect.

In summary, our study provides a comprehensive analysis of the distribution of TIM-1 in the TME and investigates the prognostic value of TIM-1+immune cells using mIF staining. Beyond validating the prognostic significance of TIM-1+B cells, we also explored two mechanisms through which TIM-1+B cells impact prognosis. This study lays the foundation for future research and the development of novel anti-tumor therapies, contributing to the advancement of precision oncology.

## Data Availability

The raw data supporting the conclusions of this article will be made available by the authors, without undue reservation.
