# Supplementary material for: Distribution characteristics and prognostic value of TIM-1 in patients with lung adenocarcinoma
Source: Front Immunol. 2025 May 30;16:1602868. doi: 10.3389/fimmu.2025.1602868 (PMC12162315; doi:10.3389/fimmu.2025.1602868)
Supplement: Supplementary file 1 [file DataSheet1.docx]

Table S1 The cut-off value of each cell type

| Cell type | Cut-off value | 95% CI |
| --- | --- | --- |
| TIM-1+CD4+T cell in TUMOR | 0.0205269 | 44.89 to 86.56 |
| TIM-1+CD8+T cell in TUMOR | 0.0008544 | 45.72 to 68.82 |
| TIM-1+B cell in TUMOR | 0.09670943 | 65.33 to 85.44 |
| TIM-1+CD4+T cell in TDLN | 0.0026715 | 38.44 to 78.55 |
| TIM-1+CD8+T cell in TDLN | 0.0147904 | 67.97 to 103.80 |
| TIM-1+B cell in TDLN | 0.1463774 | 50.16 to 96.61 |
| CD8+T cell in TUMOR | 0.0386763 | 55.16 to 77.58 |

**Figure S1** Time-dependent ROC curves and Kaplan–Meier survival analysis of different TIM-1 positive cell type. The time-dependent ROC curves for TIM-1+CD4+T cell in the tumor (A), TIM-1+CD8+T cell in the tumor(D), TIM-1+CD4+T cell in the TDLN(G), TIM-1+CD8+T cell in the TDLN(J). TIM-1+CD4+T cell in the tumor was not related to the OS (B) and DFS (C) of LUAD patients. TIM-1+CD8+T cell in the tumor was not related to the OS (E) and DFS (F) of LUAD patients. TIM-1+CD4+T cell in the TDLN was not related to the OS (H) and DFS (I) of LUAD patients. TIM-1+CD8+T cell in the TDLN was not related to the OS (K) and DFS (J) of LUAD patients.

**Figure S2** Kaplan–Meier survival analysis of TIM-1+B cell in the validation cohort. TIM-1+B cell in the tumor was not related to the DFS (A). TIM-1+B cell in the tumor was related to the OS (B). TIM-1+B cell in the TDLN was related to the DFS(C) and OS (D).

**Figure S3** Correlation analysis of TIM-1+B cell / B cell and the number of TLS. (A)TIM-1+B cell / B cell in the TUMOR was not related to the number of TLS. (B)TIM-1+B cell / B cell in the TDLN was not related to the number of TLS. (C) High density of TIM-1+B cell in the tumor primary lesion was associated to the low density of CD8+T cell. (D) TIM-1+B cell in the tumor primary lesion was not associated to the infiltration of CD4+T cell. (E) TIM-1+B cell in the TDLN was not associated to the infiltration of CD4+T cell. (F) TIM-1+B cell in the TDLN was not associated to the infiltration of CD8+T cell.
